# Modeling the Glomerular Filtration Barrier and Intercellular Crosstalk

**DOI:** 10.3389/fphys.2021.689083

**Published:** 2021-06-02

**Authors:** Kerstin Ebefors, Emelie Lassén, Nanditha Anandakrishnan, Evren U. Azeloglu, Ilse S. Daehn

**Affiliations:** ^1^Department of Physiology, Institute of Neuroscience and Physiology, Sahlgrenska Academy, University of Gothenburg, Gothenburg, Sweden; ^2^Division of Nephrology, Department of Medicine, Icahn School of Medicine at Mount Sinai, New York, NY, United States

**Keywords:** glomerular filtration barrier, crosstalk, *in vitro*, podocyte, glomerular endothelial cell, 3D model

## Abstract

The glomerulus is a compact cluster of capillaries responsible for blood filtration and initiating urine production in the renal nephrons. A trilaminar structure in the capillary wall forms the glomerular filtration barrier (GFB), composed of glycocalyx-enriched and fenestrated endothelial cells adhering to the glomerular basement membrane and specialized visceral epithelial cells, podocytes, forming the outermost layer with a molecular slit diaphragm between their interdigitating foot processes. The unique dynamic and selective nature of blood filtration to produce urine requires the functionality of each of the GFB components, and hence, mimicking the glomerular filter *in vitro* has been challenging, though critical for various research applications and drug screening. Research efforts in the past few years have transformed our understanding of the structure and multifaceted roles of the cells and their intricate crosstalk in development and disease pathogenesis. In this review, we present a new wave of technologies that include glomerulus-on-a-chip, three-dimensional microfluidic models, and organoids all promising to improve our understanding of glomerular biology and to enable the development of GFB-targeted therapies. Here, we also outline the challenges and the opportunities of these emerging biomimetic systems that aim to recapitulate the complex glomerular filter, and the evolving perspectives on the sophisticated repertoire of cellular signaling that comprise the glomerular milieu.

“A model is a lie that helps you see the truth” – Dr. Howard Skipper

## Introduction

The glomerular filtration barrier (GFB) is a highly specialized interface responsible for blood filtration that is charge and size selective. While its functionality and integrity are maintained by a constant interaction between glomerular endothelial cells (GECs), the glomerular basement membrane (GBM), and podocytes (Rennke et al., 1975; Rennke and Venkatachalam, 1979), they are also influenced by the milieu and dynamics of the renal blood flow. In glomerular diseases, this barrier loses functional integrity, allowing the passage of macromolecules and cells, and results in morphological changes, increasing the risk of long-term kidney damage that ultimately leads to kidney failure (USRDS, 2020). This is a growing worldwide health problem that accounts for a substantial economic burden (Honeycutt et al., 2013). Although the etiologies differ among glomerular diseases, damage to the GFB often has the same clinical manifestations, proteinuria or hematuria, and impaired glomerular filtration rate (GFR).

The interconnectivity and structural complexity of the GFB have favored the use of experimental *in vivo* models, where these traits are preserved. Using rodent models is regarded as the gold standard in GFB research. Mice have been used extensively to study the GFB, given the advantage that the complexity of the GFB microenvironment can be fully recapitulated, that there are several available genetically defined strains and the relative ease of single gene targeting (Becker and Hewitson, 2013). Also, transgenic lines with fluorescent reporters in different glomerular cell types provide visual readout and have been useful for determining the origins and fate of glomerular cells *in vivo* (Hackl et al., 2013). There are however significant challenges with mimicking human disease in animals, as many models do not completely recapitulate human disease manifestations and instead allow only for studies of certain disease aspects (Becker and Hewitson, 2013). However, the use of animal models is of particular importance for pharmacodynamics and pharmacokinetics testing, where the effects of pharmaceutical interventions can be examined at the systemic level to determine drug safety and efficacy before entering human trials.

The use of transgenic zebrafish strains is growing as a vertebrate model for GFB research (Zhou and Hildebrandt, 2012; Hansen et al., 2020) and has proven to be a useful tool to investigate glomerular disease development and the effects of drugs on GFB (Schiffer et al., 2015; Müller-Deile et al., 2019). Although studies in zebrafish are more time- and cost-efficient compared with rodent models, there are some inherent caveats. It can for instance be difficult to detect proteinuria or the clearance of specific markers of interest in the urine due to the surrounding water volume. Also, zebrafish have numerous duplicate genes (Woods et al., 2000), which complicates the generation of knockout strains, and they also have the ability to regenerate nephrons de novo after injury. Other limitations include the need for microinjections to the dorsal aorta and cardinal vein for certain drugs, which limits throughput. Altogether, animal work can be expensive, has limited throughput, and poses challenges for studying intricate crosstalk between the cells in the glomerulus. Therefore, there is a need for microphysiological systems that can recapitulate the form and function of the GFB and offer a controlled environment for studies of isolated pathological events. Current model systems range from simple to physiologically complex and offer opportunities for examining specific mechanisms involved in the maintenance as well as damage to the GFB (Table 1). Here, we review and discuss some of the current and future experimental *in vitro* model systems for studying the GFB.

**Table 1:** Comparison of *in vivo* and *in vitro* models currently used or under development for studies of the glomerular filtration barrier (GFB).

	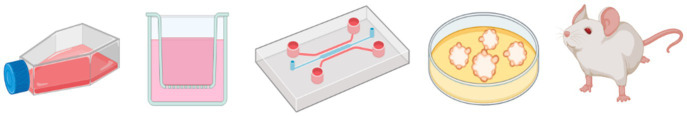				
	*In vitro*				*In vivo*
	2D monolayer	Static co-culture	Microfluidic co-culture	Spheroids organoids	Animal models
All GFB cell types (Nishinakamura, 2019)	No	No	No	No	Yes
GBM (Slater et al., 2011; Chew and Lennon, 2018; Hale et al., 2018; Petrosyan et al., 2019)	No	Limited	Limited	Limited	Yes
Glycocalyx (Singh et al., 2007; Petrosyan et al., 2019; Koning et al., 2020)	Limited	Limited	Yes	Limited	Yes
Allows cell differentiation (relevant phenotype; Musah et al., 2017; Bao et al., 2018; Nishinakamura, 2019; Veissi et al., 2020)	No	No	Limited	Limited	Yes
Permselectivity (Li et al., 2016; Petrosyan et al., 2019; Li et al., 2020b)	No	Yes	Yes	Limited	Yes
Recapitulation of microenvironment (Huh et al., 2013; Bhatia and Ingber, 2014; Veissi et al., 2020)	No	Limited	Limited	Limited	Yes
Controlled microenvironment (Anandakrishnan and Azeloglu, 2020)	Yes	Yes	Yes	Yes	No
Shear stress (Slater et al., 2012; Musah et al., 2017; Yang et al., 2017; Homan et al., 2019)	No	Limited	Yes	Limited	Yes
Bidirectional crosstalk (Li et al., 2016; Casalena et al., 2020; Veissi et al., 2020)	No	Yes	Yes	Limited	Yes
Material of human origin (Little and Takasato, 2015; Musah et al., 2017; Anandakrishnan and Azeloglu, 2020)	Yes	Yes	Yes	Yes	No
High throughput (Boreström et al., 2018; Anandakrishnan and Azeloglu, 2020)	Yes	Limited	Limited	Limited	No
Development of personalized/precision medicine (Anandakrishnan and Azeloglu, 2020)	Yes	Yes	Yes	Yes	No
Timeline for experiment	Short	Short	Long	Long	Long

## The Functional Barrier

**Figure 1:**
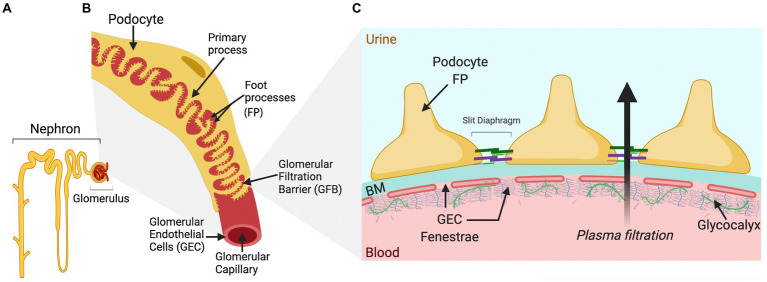
Schematic drawing of a single nephron and glomerulus, a glomerular capillary vessel, and the glomerular filtration barrier (GFB). **(A)** A single nephron comprising the blood-filtering glomerulus, enveloped by Bowman’s capsule that connects to the proximal tubule at the start of the urine-modifying tubular system. **(B)** The luminal surface of the glomerular capillary vessel is covered by glomerular endothelial cells (GECs), while podocytes wrap around the outside of the vessel with primary and foot processes (FT), forming an interdigitated pattern. Neighboring FPs are bridged by the slit diaphragm, one of the several essential components for glomerular permselectivity. The blood is filtered over this capillary barrier, and the pre-urine produced is forwarded from the Bowman’s capsule into the lumen of the proximal tubule. **(C)** The schematic cross section of the GFB displays the GEC fenestrae attached to the basement membrane (BM) and covered luminally by glycoproteins, proteoglycans, and glycosaminoglycans of the glycocalyx, important for maintaining the charge selectivity of the GFB. On the opposite side of the BM, the podocyte FPs are attached. The FPs interlink by slit diaphragm proteins, such as nephrin and podocin, are important for the restriction of albumin by the GFB. The arrow shows the direction of plasma filtration over the barrier.

The glomerulus is the filtering part of the nephron (Figure 1A) and consists of three different cell types: podocytes (visceral epithelial cells), GECs, and mesangial cells. The filtrate from the glomerulus enters the Bowman’s capsule as pre-urine before reabsorption and secretion in the tubular system. Glomerular cells are highly specialized and interdependent, with fenestrated GECs covering the luminal surface of glomerular capillaries, in direct contact with the blood. Podocytes tightly wrap around the glomerular capillary vessels, with interdigitating foot processes bridged by a slit diaphragm (Figure 1B). GECs and podocytes share a common extracellular matrix (ECM), the glomerular basement membrane (GBM), and together, they form the GFB (Figure 1C). Between the capillaries are contractile mesangial cells surrounded by their ECM, providing structural support to the glomerular tuft (Brenner et al., 1978).

The GFB function relies on its three layers: podocytes, GBM, and GECs (Figure 1C). Podocytes are terminally differentiated epithelial cells that form the architectural backbone of the GFB anchored to the GBM through transmembrane receptors, such as integrins (e.g., integrin α3 and laminin β2) and dystroglycan, and cover the outer aspect of the glomerular capillary (Pozzi et al., 2008; Meyrier, 2011). They have specialized projections that interdigitate to form the slit diaphragm, a key element in the GFB (Perico et al., 2016). The slit diaphragm proteins (e.g., nephrin and podocin) anchor to the cytoskeleton at the plasma membrane and form bridging structures between the interdigitating podocyte projections (foot processes; Kestila et al., 1998; Boute et al., 2000). Additional proteins that maintain slit diaphragm proteins, such as CD2AP, play vital roles in GFB maintenance. Podocytes are essential in GFB function, underscored by the discovery of pathogenic mutations to proteins involved in maintaining podocyte structure that are causal to proteinuric forms of kidney disease (Vivante and Hildebrandt, 2016; Li et al., 2020a). The GBM is formed by secreted products from both podocytes and endothelial cells during glomerulogenesis (St John and Abrahamson, 2001). Its role in the barrier function is highlighted by genetic studies showing that mutations in key components of GBM; encoding laminin-α5 and *COL4A5*, or recessive *COL4A3/4*, results in basement membrane nephropathy due to the absence or inadequate assembly of all collagen chains. These mutations contribute to the development of nephrotic syndrome in pediatric patients and Alport syndrome, respectively (Tryggvason et al., 1993; Kashtan, 1999; Quinlan and Rheault, 2021).

Glomerular endothelial cells are highly specialized cells with fenestrae and a charged luminal endothelial surface layer, or glycocalyx, that is composed of negatively charged networks of proteoglycans, glycoproteins, and glycolipids (Ballermann, 2007; Fogo and Kon, 2010; Haraldsson and Nystrom, 2012; Khramova et al., 2021) that together with the GBM contribute to the maintenance of a charge-selective barrier which is important to restrain albumin from the glomerular filtrate (Jeansson et al., 2009; Singh et al., 2011; Öberg and Rippe, 2013; Boels et al., 2016; Figure 1C). GEC dysfunction can initiate and contribute to GFB breakdown (Haraldsson et al., 2008; Haraldsson and Nystrom, 2012; Sun et al., 2013; Daehn, 2018). In addition, activated podocytes have been shown to influence endothelial glycocalyx remodeling and loss in experimental FSGS and *in vitro* (Ebefors et al., 2019). In diabetic kidney disease, GEC dysfunction and glycocalyx damage represent initiating steps in diabetic albuminuria in humans and in experimental models (Zhao et al., 2006; Satchell and Tooke, 2008; Yuen et al., 2012; Dogne et al., 2016; Lassen and Daehn, 2020).

Importantly, bidirectional signaling enables cells in the glomeruli to function effectively, where podocytes control GEC growth and survival *via* crosstalk of paracrine vascular endothelial growth factor alpha (VEGFA and VEGF-R; Sison et al., 2010; Jeansson et al., 2011). Crosstalk also exists between endothelial and mesangial cells (PDGF-B and PDGFR-β) and between podocytes and mesangial cells (CCL21 and CCR7; Vaughan and Quaggin, 2008; Schlondorff and Banas, 2009). Hence, all components contribute to the overall structure and function of this complex barrier, and model systems that can recapitulate *in vivo* biology and microenvironment would provide a platform for studying cell crosstalk and feedback regulation and open up the new therapeutic strategies specifically targeting the GFB.

### Modeling the Glomerular Filtration Barrier

The unique environment and complex interactions between the specialized cells in the GFB make modeling glomerular disease particularly challenging. Podocytes are a key target cell for injury in the evolution of segmental sclerosis lesions of proteinuric diseases, and their morphology is critical for glomerular filtration. However, once isolated, podocytes rapidly dedifferentiate and lose their specialized morphology, making it difficult to study their function *in vitro*. Immortalized mouse and human podocyte cell lines have played a fundamental role in advancing podocyte research, but they lack defined foot processes as well as slit diaphragms. Efforts have been made to improve podocytes in culture to more closely recapitulate their *in vivo* phenotypic characteristics. By modulating the ECM, which affects most aspects of cellular behavior, researchers have established that growing primary rat podocytes in the presence of heparin and all-trans retinoic acid on laminin-coated plates resulted in podocytes with primary processes that further bifurcated and interdigitated with adjacent cells (Yaoita et al., 2018). Growing podocytes in a gelatin microbial transglutaminase platform tuned to the stiffness of healthy glomeruli promoted the differentiation and maturation response of podocytes (Hu et al., 2017). Other approaches involve culturing podocytes on nanoporous surfaces with grooves. This method showed that podocytes were better differentiated, had organized actin cytoskeleton stress fibers, and developed vinculin-positive focal adhesions (Zennaro et al., 2016). Microscale curvature surfaces have also been shown to promote podocyte differentiation *in vitro* (Korolj et al., 2018). By growing podocytes on topographic substrates, the authors showed augmented nephrin expression and structured F-actin arrangement within cells. The curved surfaces promoted process formation with interdigitation and improved barrier function compared to podocytes grown on flat substrates (Korolj et al., 2018). Bioengineered surfaces that artificially induce branch formation have been developed by growing podocytes on a 3D geometry that mechanically enforces the arborization of individual podocytes (Ron et al., 2017). The formation of peripheral projections showed increased slit diaphragm proteins (nephrin, podocin, and NEPH1) and synaptopodin, as well as actinin-4 cross-linked actin stress fibers properly localized within these peripheral processes. In addition to observing slit diaphragm-like cell-cell junctions, the authors also demonstrated that on these surfaces, podocytes had a significant increase in expression of genes related to podocyte function, hence a more mature physiological phenotype (Ron et al., 2017). The next steps are already underway involving the derivation and generation of human pluripotent stem cells into podocyte-like cells (Yaoita et al., 2018; Ge et al., 2020). These will be instrumental for future studies and high-content screening for podocentric therapies, and for integration into more complex model systems discussed below.

There are also challenges in obtaining, culturing, and maintaining GECs *in vitro*. GECs differ in anatomy to most other endothelial cells in the body and are defined by their fenestrations, which are important for the function of the filtration barrier (Satchell and Braet, 2009; Fogo and Kon, 2010). The fenestrations lack diaphragm but are covered with a glycocalyx. Mimicking GEC function *in vitro* has been challenging as they lose fenestrations in culture. This may be due to their dependence on podocyte-derived growth factors for their viability through intercellular crosstalk and interactions with the GBM. However, the very first human glomerular endothelial cell (GEnC) line, developed by Satchell et al. (2006), was shown to have fenestrations in response to VEGF, and over the years, it has proved to be a useful tool in GFB research, including in studies of glomerular cell interactions (Boor et al., 2010; Byron et al., 2014). The importance of VEGF-C on GEC monolayer permeability has been demonstrated through the measurement of trans-endothelial electrical resistance (TEER) as an indicator of the integrity of GEC’s intercellular junctions (Ramnath and Satchell, 2020) and the passage of fluorescence-labeled BSA (Foster et al., 2008). The authors found that VEGF-C increased TEER and limited albumin passage, in contrast to the effect of VEGF-A, suggesting that these podocyte-derived growth factors regulate the permeability of GECs in the GFB (Foster et al., 2008). Although quantification of the glomerular endothelial glycocalyx *in vivo* has been achieved by direct labeling or indirect measurements (Hjalmarsson et al., 2004; Dane et al., 2015), measuring the glycocalyx in cultured GECs has been challenging due to the nature of this invisible layer. Recently, atomic force microscope elastography was used to successfully measure 3D biomechanical properties of the glycocalyx on murine GECs through direct contact by deflection of a cantilever, without exposing cultured cells to fixation or staining procedures that alter the fragile structure (Ebefors et al., 2019). An additional requirement of GECs function is fluid flow, which is absent in monocultures, leading to loss of the influence of shear stress on cell shape and signal transduction that is present under physiological conditions (Ballermann et al., 1998). One shear stress-inducible transcription factor is Krüppel-like factor 2 (KLF2; Lee et al., 2006), an important regulator of hemodynamic signals in endothelial cells that has been shown to be dysregulated in diabetic kidney disease. Importantly, the endothelial cell-specific knockout of KLF2 results in worsened endothelial cell and podocyte injury in an experimental model of type 1 diabetes (Zhong et al., 2014).

In addition to the challenges of providing a favorable biophysical environment for glomerular cells, ideal models of the GFB should allow for adjustment of the GFR, given that hyperfiltration occurs under physiological conditions, such as during pregnancy, and is commonly observed in DKD, polycystic kidney disease, and sickle-cell anemia (Helal et al., 2012; Cheung and Lafayette, 2013). A physiological decline in GFR is conversely associated with advancing age (Musso and Oreopoulos, 2011). Hence, adjustable GFR is an important consideration for the physiological relevance of *in vitro* GFB models that can be addressed by using microfluidic devices. Innovative tools are still needed to account for tubuloglomerular feedback (TGF) that is regulated *via* macula densa cells in the distal tubule and the myogenic response (Vallon, 2003). TGF has mostly been studied *in vivo* due to the challenges of studying the intricate signaling between these cells *in vitro*.

Despite some of the challenges mentioned, *in vitro* models are making substantial progress as an alternative or complement to *in vivo* experimental models for mechanistic studies of the GFB components and intercellular crosstalk. In the following sections, we review the recent developments in this evolving field.

## Studying Glomerular Cell Crosstalk

Two-dimensional (2D) cultures are a simple culture system to study glomerular cell-specific effects, as they provide screening of large numbers of conditions and treatments that would otherwise not be possible *in vivo* (Table 1). To study glomerular crosstalk, conditioned medium transfer is necessary when using 2D cultures. Despite the inherent limitations of 2D cultures, this system allows to chronologically separate cellular signaling events of pathogenic stimuli that ultimately lead to cell and/or organ dysfunction.

There are different strategies used for the conditioned medium transfer, and these have been well described by Hanspal et al. in the context of amyotrophic lateral sclerosis research (Hanspal et al., 2017). The simplest strategy consists of whole medium transfer from one monoculture to another in separate culture vessels. There can also be an intermediate step of extraction or enrichment of specific media components before medium transfer to the acceptor cell culture. Insights from this approach have provided evidence for the pathologic effects of the milieu in women with preeclampsia, where factors including endothelin-1 from GECs exposed to the serum from patients with preeclampsia resulted in shedding of nephrin from podocytes cell surface *via* endothelin receptor A after media transfer (Collino et al., 2008). Another study utilized the transfer of purified exosomes from high glucose-treated GECs to podocytes and found that TGFβ mRNA, carried by the extracellular vesicles, contributed to podocyte dedifferentiation epithelial-mesenchymal transition (Wu et al., 2017). The authors found the same mechanism of exosomes containing TGFβ mRNA to contribute to mesangial cell proliferation and matrix production through a similar experimental setup, as well as through tail-vain injections of the purified exosomes from high glucose-treated GECs in C57BL/6 mice (Wu et al., 2016). Furthermore, the studies of TGFβ-containing exosomes by another group supported the involvement of these extracellular vesicles in glomerular crosstalk following high glucose stimulation (Wang et al., 2018b). Exosomes have emerged as a novel vector for cell-cell communication in the kidney, and they are beginning to be recognized more and more as a critical player in the pathogenesis of kidney disease and decline in renal function.

Co-culture of two or more cell types offers increased complexity over monocultures when studying glomerular crosstalk. Open microfluidics systems allow simultaneous paracrine signaling between two separated cell populations by sharing culture medium and hence allow for exchange of soluble factors and transient signals (Zhang et al., 2020). In transwell systems, two distinct cell types are separated by a porous membrane (Hanspal et al., 2017), where a bidirectional exchange of signaling molecules can occur with or without direct cell-cell contact (Table 1). Li and colleagues demonstrated the applicability of their co-culture model of the GFB for studies of drug testing and intracellular signaling, using murine podocytes and GECs on opposite sides of a collagen IV-coated polyethylene terephthalate membrane (Li et al., 2016). More recently, the same research group successfully exchanged the murine glomerular cells for human immortalized GECs and podocytes, and reported an increase in albumin leak after exposure to sera from patients with recurrent FSGS, compared to genetic or non-recurrent forms (Li et al., 2020b). Casalena et al. have demonstrated that both high glucose and serum from diabetic mice susceptible to developing diabetic kidney disease disrupt mitochondrial function and cause oxidative stress in GECs. Interestingly, the transfer of factors released by the stressed GECs mediated podocyte cell death in transwell co-cultures, as well as in media exchange (Casalena et al., 2020). Given that bi-directional communication can still occur while cells are physically separated, this approach allows for subsequent interrogation of cell-specific responses. This approach has also been used to define podocyte-to-GEC-to-podocyte crosstalk in the pathogenesis of FSGS by shedding light on the role molecules, such as endothelin-1/endothelin receptor type A-mediated glomerular endothelial cell dysfunction, which was shown to be required for podocyte depletion and progression of glomerulosclerosis (Daehn et al., 2014).

Exposure of GECs to laminar shear forces found *in vivo* adds physiological relevance to the transwell co-culture model of the GFB. Studies by Slater et al. used both conditioned medium transfer and co-culture of human GECs and podocytes to investigate how ERK5 activation and KLF2 transcription (associated with endothelial cell shear stress in large vessels) affected the glomerular microvasculature (Slater et al., 2012). Their findings demonstrated the existence of intercellular signaling from GECs exposed to chronic laminar shear stress that affects podocytes. In another study by the same research group, GECs and podocytes were co-cultured on opposite sides of a polycaprolactone/electrospun collagen membrane to closer mimic the GBM, which was shown to enable cell-cell contact (Slater et al., 2011). Differences in between the conditioned medium transfer and the co-culture settings suggest that spatial separation between crosstalking cell types is an important consideration.

The models described so far provide robust high-throughput, high-content reductionist assay systems. They have provided a wealth of information on the fundamental biological and disease processes of the GFB. Nevertheless, they provide a limited physiological context of the filtration barrier. Since there is growing awareness of the interconnections between cells and the ECM surrounding them, there is substantial effort by the community to develop model systems that can better reflect the complex microenvironment cells encounter in a tissue.

### 3D Culture Models of the GFB

Organs-on-a-chip have been developed for complex organs such as liver (Beckwitt et al., 2018), heart (Agarwal et al., 2013), gut (Kim et al., 2012; Kim and Ingber, 2013), lungs (Huh et al., 2010, 2012), and brain (Moreno et al., 2015). The goal has not been to mimic the whole organs, but rather to study complex parts of an organ in a more physiological context. In the renal field, chips for modeling the proximal tubules (Jang et al., 2013; Hoppensack et al., 2014; Wilmer et al., 2016) as well as the filtration barrier are being developed. An ideal model of the GFB would include cell-to-cell and cell-to-ECM interactions, biomimetic micromechanical properties, shear flow, oxygen and nutrient/waste exchange, and a functional permselective filtration barrier. In the last decade, the development of microfluidic platforms that allow co-culture of cells under flow (Bhatia and Ingber, 2014) and stretch (Huh et al., 2013) has emerged (Table 1) and these continue to evolve. Here, we describe some examples.

To study the effect of hypertension on the filtration barrier, Zhou et al. developed a glomerulus-on-a-chip using murine immortalized GECs and podocytes. The cells were separated in the chip by a polycarbonate membrane coated with basement membrane extracts, and the authors increased the flow in the upper channel of the chip harboring the GECs (Zhou et al., 2016). Increasing the mechanical force led to cell damage, loss of junctions, and changes to the cell’s cytoskeleton, leading to increased leakage (Zhou et al., 2016). In an *in vitro* model of diabetic kidney disease, Wang et al. developed a glomerulus-on-a-chip using glomeruli isolated from rats. The chip consisted of five channels, a capillary in the middle and collection channels on the outside, with the channels in between filled with gel. Isolated glomeruli were injected in the capillary channel and allowed to attach for the cells to spread and form a barrier under flow. GECs and podocytes were identified by CD31 and synaptopodin staining, respectively. High glucose treatment enhanced the permeability to proteins and increased reactive oxygen species production and podocyte detachment (Wang et al., 2017). Musah et al. developed a glomerulus-on-a-chip with fluidics and strain by using vacuum channels on the side of the channel harboring the GECs and podocytes (Musah et al., 2017). The authors developed podocytes derived from human induced pluripotent stem cells (iPSCs) and used them in combination with human GECs separated by a porous polydimethylsiloxane membrane coated with laminin. The mechanical strain was shown to increase the expression of nephrin and secretion of VEGF-A by the podocytes. Albuminuria and podocyte damage were observed with adriamycin treatment, underscoring the resemblance to the *in vivo* setting (Musah et al., 2017). These models however lack GBM; hence, Petrosyan et al. developed a glomerulus-on-a-chip without an artificial membrane between GECs and podocytes (Petrosyan et al., 2019). The authors allowed both cell types to interact and to generate a layer of ECM components. Human GECs and podocytes were obtained from the same donor; cells were separated by collagen I and eventually formed a basement membrane between the cell layers. GECs were further shown to develop a glycocalyx layer. The cells could be maintained in the chip for at least a month, enabling long-term experiments. Exposure of chips to puromycin aminonucleoside induced podocyte injury and loss of permselectivity for albumin. Adding serum from patients with membranous nephropathy (MN) resulted in albumin leakage, which was prevented by treatment with α-MSH. Using podocytes derived from a patient with Alport syndrome rendered improper filtration, supporting the chips potential for the use in personalized medicine (Petrosyan et al., 2019).

Given that the glomerulus *in situ* has a complex structure with intricate microvascular capillary networks in a unique geometry that could play a role in the development and function of podocytes (Falkenberg et al., 2017), there have been significant efforts to generate 3D models with complex microvascular networks using 3D bioprinting technology. Rayner et al. demonstrated the use of a multiphoton microscopy-guided 3D printing technique to generate perfusable vascular networks with diameters as small as 10 μm (Rayner et al., 2021). They further demonstrate bioprinting of a glomerular-like microvascular network that supports endothelial lumen formation; however, they still require the incorporation of podocytes and mesangial cells to recapitulate the glomerular physiology and to study cell-cell crosstalk. Other developments include the glomerulus-on-a-plate, recently developed by using a microfluidic topographical hollow fiber (Xie et al., 2020). This system uses a tubular-like perfusable channel to seed GECs in a glomerulus-like knot with microconvex topography, filled with hydrogel and covered with murine podocytes. The fibers were mounted in specialized 96-well plates with inlet and outlet wells allowing flow to be applied by either gravity or syringe pump. Perfusing the lumen with albumin showed no leakage of over the barrier, while small molecules could readily pass. However, adriamycin treatment was shown to increase the passage of BSA over the barrier, but only mildly damaged podocytes (Xie et al., 2020).

Current GFB 3D culture model technologies have a number of drawbacks, such as recirculating instead of a continuous flow, long culture times to achieve fully confluent layers, lack of a basement membrane, and limited throughput. However, these models still hold great promise for improving our understanding of glomerular crosstalk and their potential use for personalized and precision medicine. In the future, chips where cells can form a basement membrane without separating gels or man-made membranes will emerge, and the inclusion of mesangial cells, pericytes, and parietal epithelial cells to the chips would enable all the intricate signaling which takes place in the glomerulus.

### Scaffold-Free 3D Cultures

Scaffold-free 3D cultures are anchorage-independent models that rely on the self-aggregation of cells in specialized culture plates with ultra-low attachment coating that promotes spheroid formation. Multicellular spheroids have been shown to recapitulate physiological characteristics of tissues and tumors with regard to cell-cell contact, and allow for natural cell-ECM interactions (Sutherland, 1988). Glomeruloid spheres have been developed using human mesenchymal stem cells, HUVECs, and HEKs (Abe et al., 2019). These spheroids expressed several podocyte markers and were stable for at least 5 days. Adding serum from patients with FSGS resulted in the collapse of the spheres (Abe et al., 2019). In 2020, Cho et al. demonstrated a novel pressure-assisted network for droplet accumulation method for high-throughput generation of uniform microtissues. As a proof of principle, they generated glomerulus-like microtissues using immortalized mouse podocytes and mesenchymal stem cells (Cho et al., 2020). More recently, Sobreiro-Almeida et al. observed that the addition of retinoic acid to an organotypic model of human renal progenitor cells resulted in spheroids with a preferential glomerular differentiation. Using a hanging drop culture technique to form spheroids, they showed that these spheroids remain viable over a period of 28 days and display an elevated expression of *PAX2* and *NPHS1* in the presence of retinoic acid. Further, co-culture with microvascular endothelial cells resulted in more compact organization of the spheroids (Sobreiro-Almeida et al., 2021).

These scaffold-free 3D cultures are not barrier models, and many questions remain: in particular, about the composition of the spheres. And improvement in oxygenation through integration of endothelial cells has not been examined in this setting. Today’s glomeruloid spheres can provide insights for podocyte-ECM interactions and can be adapted to medium- or high-throughput screening assays. There is still the need for culture optimization to enhance reproducibility of spheroids in culture and to study GFB components, while maintaining a small enough size for sufficient nutrient exchange. However, this area of research is moving fast, and we will undoubtedly see advances in the years to come.

### Organoids

Attempts to fully culture organs *in vitro* have led to the development of organoids, self-organized 3D aggregations of cells. Over the last few years, these developments have provided researchers the opportunity to establish near-physiological models to study human development and diseases. Organoids can be derived from embryonic stem cells or iPSCs. The kidney is an anatomically complex organ with numerous different cell types, which makes it difficult to get organoids containing all renal structures including a functional filtration barrier. As of today, organoids are premature, and as such, they do not represent ideal modeling systems for studies of the GFB; however, they hold promise to be so in the future.

Embryonic kidneys are divided into the metanephric mesenchyme and the ureteric bud. Nephron progenitor cells in the metanephric mesenchyme are the origin of the glomeruli, Bowman’s capsule, and the renal tubules, and stromal progenitor cells give rise to interstitial cells. The ureteric bud is the origin of the collecting ducts. During development, intricate signaling leads to differentiation of cells and the formation of a mature kidney. In order to form kidney organoids, this signaling needs to be applied to embryonic or pluripotent stem cells. With this in mind, the development of differentiation protocols for embryonic and iPSCs toward renal cells (Xia et al., 2013; Taguchi et al., 2014; Takasato et al., 2014) was rapidly followed by the first reports of kidney organoids (Morizane et al., 2015; Takasato et al., 2015). Kidney organoids have been characterized *via* single-cell sequencing and have been found to contain developing podocytes, parietal epithelial cells, tubular cells, collecting ducts, and interstitial and stromal cells. Missing or underrepresented cells with current methods are GECs, mesangial cells, principal and intercalated cells (Czerniecki et al., 2018; Wu et al., 2018; Combes et al., 2019), and immune cells. Although glomerulus-like structures are formed, they mainly consist of early podocytes, and these have the potential to be explored further to study podocytopathies (Sharmin et al., 2016; Kim et al., 2017; Hale et al., 2018). Hale et al. describe a protocol for kidney organoids from iPSCs and compared the expression to human immortalized podocyte cell lines. Podocytes derived from organoids were shown to have an improved expression profile, as well as a GBM (Hale et al., 2018). Genetic modifications targeting podocytes have also been used in kidney organoids to explore congenital nephrotic syndrome (Kim et al., 2017; Hale et al., 2018; Tanigawa et al., 2018). In addition, to better study the GFB, improvements in methods that promote maturation and vascularization of the organoids have been reported recently, such as culturing kidney organoids on millifluidic chips (Homan et al., 2019), or transplantation of human kidney organoids into the subcapsular of mouse kidneys (van den Berg et al., 2018). In the latter, the authors demonstrated an improvement in the formation of a GBM with the development of a fenestrated endothelium in glomeruli (van den Berg et al., 2018). By modulating biophysical cues, such as ECM stiffness, Garreta et al. were able to accelerate kidney organoid generation from iPSCs (Garreta et al., 2019). They showed that implantation of kidney organoids into chick chorioallantoic membrane (CAM) resulted in vascularization of the organoids within 5 days. They further generated soft hydrogels that display similar mechanical properties as CAM to study if soft substrates drive kidney organoid generation compared to stiffer substrates. They observed that soft matrix environment resulted in kidney organoids that display similar protein expression as a fetal human kidney. Although the kidney organoids still are embryonic in development and need an *in vivo* environment for vascularization, further characterization of the role of substrate stiffness can improve kidney organoid differentiation. Another limitation of the current organoid systems is the heterogeneity and batch-to-batch variation during initial formation and maturation. To address this, Dr. Little’s group have employed two different approaches for scaling up the generation of kidney organoids with less heterogeneity and higher reproducibility. Kumar et al. demonstrated a method to scale up the generation of kidney micro-organoids in suspension culture (Kumar et al., 2019). Using this method, they were able to generate 8,000–10,000 kidney micro-organoids in an even size range. These organoids are less than 200–300 μm in final size, much smaller compared to standard organoids, which allows efficient nutrient diffusion to the core of the organoids. However, they showed limited utility with respect to extended long-term cultures due to the absence of vascularization. Lawlor et al. employed extrusion bioprinting method to plate cell aggregates that mature into kidney organoids, which partially eliminates organoid heterogeneity and enables scaling up of throughput (Lawlor et al., 2021). Using this technique, they were able to generate 200 organoids in 10 min. In addition to reducing variability, extrusion bioprinting can also be used to alter the conformation of the organoids, to generate a spheroid or a rectangular cell aggregate patch based on the extruding tip movement. The authors observe that the rectangular conformation yielded a greater number of nephron units compared to the spheroid conformation (Lawlor et al., 2021), which with further improvements may be useful for the development of transplantable kidney tissues.

Despite the many challenges that still remain for organoids to fully resemble mature human kidneys, including less off targets cells as described in detail in the review by Geuens et al. (2020), organoid biobanks as repository for drug screening and development are emerging (Calandrini et al., 2020) and have the potential for applications in precision medicine.

## Future Perspective

The lack of specific treatments for diseases of the GFB is a worldwide health issue. The need for new explorative *in vitro* models is paramount to elucidate the intricate signaling of cells in the GFB. Today, there is greater recognition that components of the GFB work as an integrated functional unit. As more and more new tools become available, such as iPSCs in culture and 3D model systems, we shall look to integrate these human-relevant *in vitro* models with data-driven and mechanistic modeling as well as artificial intelligence-driven methods that can assist with *in silico* drug discovery and modeling (Azeloglu et al., 2014), which will inevitably streamline time-consuming and costly experiments. As we gain our understanding on other aspects that influence GFB function, such as tubuloglomerular crosstalk (Tasnim and Zink, 2012; Wang et al., 2018a), opportunities to “plug-in” modules will provide insights from the whole nephron’s perspective and even distant organ crosstalk. Together with the increasingly quantitative precision medicine approaches that can collate and combine clinical data with genomic information, these joint efforts can help guide the design of novel drug candidates and move the field toward the common goal of treating patients with better therapies for diseases of the GFB.

## Conclusion

As these experimental model systems continue to evolve and improve in terms of their physiological context and throughput, model systems have a huge potential to help unravel the molecular mechanisms of GFB breakdown and the pathogenic crosstalk signaling that may drive disease. These developments should minimize the use of animal models and accelerate discoveries by enabling the platforms for personalized and precision medicine to lower drug-induced adverse events, and identify new targets for treatments of kidney diseases that affect the filtration barrier.

## Author Contributions

Conceptualization by KE, EL and ID. KE, EL, NA, EA and ID wrote the manuscript. All authors contributed to the article and approved the submitted version.

### Conflict of Interest

The authors declare that the research was conducted in the absence of any commercial or financial relationships that could be construed as a potential conflict of interest.
